# Copy Number Variations in Pancreatic Cancer: From Biological Significance to Clinical Utility

**DOI:** 10.3390/ijms25010391

**Published:** 2023-12-27

**Authors:** Daisy J. A. Oketch, Matteo Giulietti, Francesco Piva

**Affiliations:** Department of Specialistic Clinical and Odontostomatological Sciences, Polytechnic University of Marche, 60131 Ancona, Italy

**Keywords:** pancreatic ductal adenocarcinoma (PDAC), copy number variations (CNVs), non-allelic homologous recombination (NAHR), patient stratification

## Abstract

Pancreatic ductal adenocarcinoma (PDAC) is the most common type of pancreatic cancer, characterized by high tumor heterogeneity and a poor prognosis. Inter- and intra-tumoral heterogeneity in PDAC is a major obstacle to effective PDAC treatment; therefore, it is highly desirable to explore the tumor heterogeneity and underlying mechanisms for the improvement of PDAC prognosis. Gene copy number variations (CNVs) are increasingly recognized as a common and heritable source of inter-individual variation in genomic sequence. In this review, we outline the origin, main characteristics, and pathological aspects of CNVs. We then describe the occurrence of CNVs in PDAC, including those that have been clearly shown to have a pathogenic role, and further highlight some key examples of their involvement in tumor development and progression. The ability to efficiently identify and analyze CNVs in tumor samples is important to support translational research and foster precision oncology, as copy number variants can be utilized to guide clinical decisions. We provide insights into understanding the CNV landscapes and the role of both somatic and germline CNVs in PDAC, which could lead to significant advances in diagnosis, prognosis, and treatment. Although there has been significant progress in this field, understanding the full contribution of CNVs to the genetic basis of PDAC will require further research, with more accurate CNV assays such as single-cell techniques and larger cohorts than have been performed to date.

## 1. Introduction

Pancreatic ductal adenocarcinoma (PDAC) is the most prevalent type of pancreatic cancer. Due to the absence of early symptoms and the lack of effective and reliable meth-ods for early diagnosis and screening, the majority of the patients (80–85%) present distant metastatic or locally advanced disease that is not resectable [1], with an overall 5-year survival rate of 12% [2]. PDAC thus remains one of the most challenging and aggressive malignancies facing oncologists today and has been projected to become the second leading cause of cancer death by 2030 [3]. A comprehensive understanding of the biology of the disease is therefore urgently needed as part of an effort to develop more effective therapy and improve survival.

Genetic variations have been appreciated since the emergence of molecular genetics. In the human genome, they are present in various forms, such as mutations, variable number of tandem repeats (VNTRs), transposable elements, structural alterations, insertion and deletion variations (indels), and single nucleotide polymorphisms/variations (SNPs/SNVs). SNPs were previously believed to be the predominant type of genomic variation responsible for most of the phenotypic variability. However, the Human Genome Project identified DNA sequence variations other than SNPs and collectively named them copy number variations (CNVs). They include translocations of various segments of a chromosome and deletions and insertions of nucleotides [4].

Among the cancer-associated genetic variations, mutations have been the best char-acterized. More recently, however, thanks to new sequencing techniques, the roles of ge-nomic recombinations, such as CNVs, in tumor onset, heterogeneity, and prognosis have also emerged [5]. For this reason, we report the involvement of CNVs in PDAC develop-ment and progression.

### 1.1. Classification of CNVs

Copy number variations refer to a phenomenon in which segments of the genome are repeated or deleted, with varying numbers of these repeats among different individuals’ genomes. Observations made in 2006, when the first comprehensive human haplotype map (HapMap) project Phase II of the human genome was constructed by Redon et al. [6], revealed that CNVs cover 12% of the human genome (about 360 Mb pairs), most of which are small-size rearrangements (<20 kb). The CNVs lay in both coding and non-coding regions, encompassing hundreds of genes and other functional elements. When the frequency of a CNV is less than 1%, it is a rare CNV, as opposed to common or polymorphic CNVs, which have a frequency >1% [7].

Researchers generally distinguish CNVs into two categories, depending on the length of the sequence affected [8]. The first category consists of copy number polymorphisms (CNPs), which are prevalent in the general population, with the majority being less than 10 kb in length and frequently enriched for genes encoding proteins that are important in immunity and drug detoxification. Therefore, these CNVs have well-documented roles in evolutionary adaptation to new environmental niches [4,9].

The second category consists of relatively rare variants that are longer than CNPs, having up to over a million base pairs. These variants, also referred to as microduplications (smaller than 5 Mb) and microdeletions [8], can arise within a family during the development of the oocyte or spermatozoa that give rise to a specific individual and be passed down to offspring.

Copy number variants have also been divided into three groups depending on their origin: (i) de novo CNVs, newly acquired but not present in a parent; (ii) germline CNVs, inherited and present in a parent; and (iii) somatic CNVs, meaning that they occurred after the single-cell stage of an embryo [10]. For example, although monozygotic (MZ) twins are expected to be genetically identical, one study on 19 pairs of MZ twins revealed many different CNVs among them and suggested that these variations may have occurred during somatic development [11]. Somatic mutations were also observed in 10–20% of the nucleated blood cells of the MZ twins [11]. CNVs have also been observed between different tissues of the same individual, further supporting the idea that CNVs can occur in either somatic or meiotic tissues [12]. Further studies on age-stratified MZ twins and single-born subjects [13] as well as on DNA samples (mainly from peripheral blood) of more than 50,000 individuals genotyped for the Gene-Environment Association Studies (GENEVA) consortium [14,15] have revealed the accumulation of CNVs with age in the nuclear genome of blood cells [13,15]. Data from population genetics analysis of CNVs and SNPs, collected in the HapMap project, showed that over 99% of the observed copy number variations of individuals are due to inheritance rather than new mutations, and nearly 80% of the former are due to common CNVs [10].

### 1.2. Mechanisms of CNV Formation

To date, several different mechanisms have been shown to be involved in the development of CNVs, including germline genomic rearrangements that result in losses or gains of DNA segments [16].

#### 1.2.1. Genomic Factors and Molecular Mechanisms of CNV Formation

**Figure 1:**
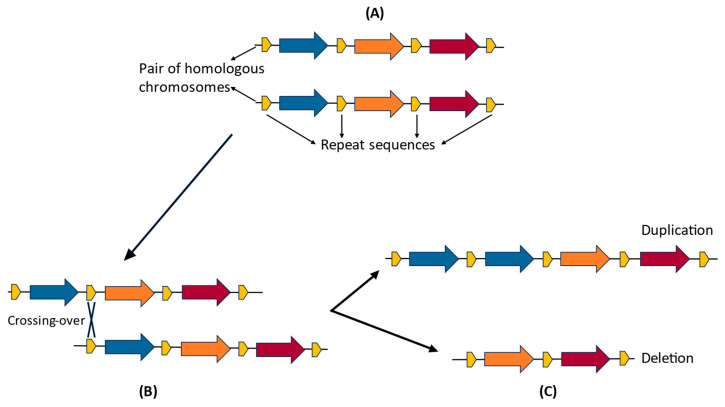
Non-allelic homologous recombination (NAHR). (**A**) Normal alignment of homologous chromosomes. (**B**) Misalignment of homologous chromosomes before crossing over, for example, due to abnormal pairing between repetitive sequences with high sequence identity. (**C**) Duplication and deletion that result from the unequal crossing over.

CNVs are produced through a variety of mutational mechanisms, including those connected to DNA replication, repair, and recombination. Although the mechanisms underlying the formation of CNVs are not completely understood, the fact that they preferentially occur within or near duplicated sequences such as long interspersed nuclear elements (LINEs) and short interspersed nuclear elements (SINEs) has provided some clues to their origin [8]. During meiosis, the presence of different repetitive DNA sequences (low copy repeats, LCRs) in male and female homologous chromosomes at non-corresponding positions (i.e., that are not alleles but share significant sequence homology) can “mislead” the recombination machinery and result in an unequal crossing-over event. This aberrant recombination, known as non-allelic homologous recombination (NAHR) [17], leads to the loss or gain of copies of genomic segments [18] (Figure 1).

Other molecular mechanisms proposed to be responsible for the formation of CNVs include the (i) replication Fork Stalling and Template Switching (FoSTeS) model [19,20], which suggests that the stalling of a replication fork can cause the lagging strand to disengage from its original template and, owing to microhomology, invade and switch to another active replication fork’s template, where it restarts DNA synthesis. The occurrence of a deletion or duplication is determined by the location of the ectopic association, and the nascent lagging strand has the potential for further disengagement and invasion of other replication forks. FoSTeS happens during DNA replication and can therefore occur either in mitosis or meiosis. (ii) Microhomology-mediated end joining (MMEJ) and non-homologous end joining (NHEJ) mechanisms [19,21], which can lead to some chromosomal rearrangements by joining nonhomologous sequences during the repair of DNA double-strand breaks (DSBs). In particular, these damages prompt NHEJ- and MMEJ-associated proteins to repair and ligate DNA sequences together. Sequence deletions, or duplications, can occur when fragments from different chromosomes are joined together. NHEJ and MMEJ occur throughout the cell cycle. Not all DSBs result in chromosomal rearrangements since they can be repaired through homologous recombination (HR) [22,23].

#### 1.2.2. Environmental Factors in CNV Formation

It is unclear how environmental factors contribute to the emergence of CNVs. However, various studies have demonstrated that chemical and physical mutagens can induce the formation of CNVs and that chemical mutagens generate copy number losses more frequently than gains, while ionizing radiation induces deletions and duplications equally across the human genome [16].

Replication stress caused by chemical mutagens, such as hydroxyurea (HU), which is a ribonucleotide-reductase inhibitor as well as an important drug for the treatment of various diseases, including sickle-cell disease, has been demonstrated to induce the formation of de novo CNVs in the human genome [24].

Physical mutagens such as ionizing radiation (including X-ray, gamma ray, and ultraviolet light) can also induce de novo CNVs through a replication-dependent mechanism because the DNA strand breaks due to radiation, which may cause the replication fork to collapse [16,25,26]. A study by Costa et al. [27] demonstrated that the offspring of parents who had exposure to low doses of ionizing radiation had a 1.5-fold higher germline CNV mutation load. In a retrospective analysis of human populations exposed to low doses of ionizing radiation, the load of de novo CNVs has been demonstrated to be a helpful biomarker of parental exposure. Another study on CNVs in papillary thyroid carcinoma (PTC) among victims of the Chernobyl accident [28] reported that following their exposure to radiation from this disaster, PTC significantly increased in the irradiated individuals. Further studies of these radiation-induced PTCs revealed more multiple aberrations of the chromosome structure than in spontaneous thyroid tumors [29,30].

### 1.3. Distribution of CNVs

Research has revealed that CNVs are more common in genes that play a role in brain development and activity and in the immune system [6], functions that have evolved rapidly in humans. In contrast, CNVs tend to be rare in genes involved in early development and basic cellular activities since the alteration of essential cellular functions can have adverse effects, suggesting that their genes could have been subjected to powerful purification selection associated with copy number variation [31].

Some scientists propose that CNVs are not random in the human genome but rather tend to cluster in areas of complex genomic architecture. These proposed hotspot regions where CNVs are enriched [20] comprise complex patterns of inverted and direct low-copy repeats (LCRs) as well as high-copy repeats (e.g., SINEs, LINEs). LCRs provide the homology required for recombination that causes NAHR-mediated modifications. LINEs and SINEs are retrotransposons that contribute to the CNVs by NAHR either because of persistent single-strandedness (e.g., due to replication pausing, secondary structures, or extensive transcription) or frequent DNA breaks in these regions (e.g., due to live transposon activity), which make them potential sites for annealing by single-stranded DNA ends [11,20].

The question of whether the localization of CNVs in the human genome is random or not is still a highly debated topic [20,32,33,34,35], but more recent studies highlight a random distribution [9,36,37].

### 1.4. Identification and Detection of CNVs

Over the years, “targeted” approaches (single gene or single panel testing) or “whole” approaches (whole genome or whole exome) have been used to detect CNVs.

#### 1.4.1. “Whole” Approaches

The process of microarray technology involves the immobilization of specific probes on a solid support, which then hybridize with target DNA segments. The two most widely used microarray technologies are array-CGH and SNP-array. In aCGH, a test sample and a reference sample are compared by labeling their genomic DNA (gDNA) with two different fluorescent dyes and applying them to an array of probes to detect differences in fluorescence intensity. On the contrary, SNP arrays consist of oligonucleotide DNA probes that correspond to regions in the genome exhibiting SNPs among individuals and do not require the use of reference sample DNA. CNV location and organization of structural variants (SVs) are not determined by microarray methods, making it necessary to subsequently perform FISH [38,39].

Next Generation Sequencing (NGS) technology involves the sequencing of highly fragmented DNA molecules to produce “reads”, which are then mapped to a human reference genome using bioinformatics software. After alignment, any differences between the newly sequenced reads and the reference genome can be identified, and the “dosage” of that specific DNA fragment and the presence of CNVs may be calculated using the number of reads generated [40]. Currently, there are four distinct methods used for the detection of CNVs from NGS data [41,42]. These methods include read-depth-based detection (RD), paired-end mapping-based detection (PE), de novo assembly-based detection (DA), and split read-based detection (SR), of which RD is the most used. Details regarding the operation of these methods have been described in other studies [43,44,45].

#### 1.4.2. “Targeted” Approaches

These involve the analysis of only one single gene or a group of genes, whether they are scattered across the genome or adjacent, and identifying a long chromosomal trait. Targeted approaches include Southern blot, fluorescent in situ hybridization, quantitative polymerase chain reaction, and multiplex ligation-dependent probe amplification.

Born in the seventies, Southern blot is still useful in the detection of some CNVs with high or extremely high numbers of repeats, particularly in the diagnosis of repeat expansion diseases. Restrictions endonucleases are used to fragment the target DNA, followed by electrophoresis to separate the resulting fragments [46]. These fragments are then incubated with DNA probes labeled either by incorporating radioactivity or by tagging the molecules with a chromogenic or fluorescent dye. CNVs are detected by comparing the hybridization intensities between a normal control and unknown samples [46] and/or by observing changes in fragment sizes (differentiated by length) and mobility following the hybridization and electrophoresis steps [39].

The fluorescent in situ hybridization (FISH) technique utilizes fluorochrome-labeled probes to match with chromosomes on a plate in order to detect any CNVs or translocations affecting a specific chromosomal region. FISH has high levels of sensitivity and specificity and is capable of detecting deletions, duplications, and translocations. However, FISH is limited in its ability to detect small imbalances and cannot be used to scan the entire karyotype without prior knowledge of the target region and appropriate probe selection. FISH can determine the location of CNVs identified by microarrays, NGS, and WGS [38,39].

Quantitative polymerase chain reaction (qPCR), also known as real-time PCR (rt-PCR), measures the accumulation of PCR amplicons in real time [39] by use of fluorescent probes. For the quantification of CNVs, a test locus with an unknown copy number and a reference locus with a known copy number are amplified in qPCR. Fluorescence intensity increases in direct proportion to the quantity of amplicon generated in each PCR cycle, and by determining the number of cycles needed to reach a specific threshold level of fluorescence, the quantity of the initial template can be determined [47].

The multiplex ligation-dependent probe amplification (MLPA) technique is based on the hybridization and ligation of specific DNA regions with two adjacently located complementary probes, followed by multiple PCR using a single pair of fluorescent primers. In particular, primer pairs containing identical 5′ sequences are used to amplify the target DNA sequences, followed by pooling into a probe mix [48] since all probes possess the same 5′ sequences. The PCR products are then separated by a capillary sequencer based on their size, and the resulting fluorescence intensities are exported for further analysis [48]. This method’s capability to analyze sequences of high identity is greatly attributed to the sensitivity of the ligation step, which allows for the design of probes containing mismatches at the ligation site. MLPA can detect CNVs at multiple loci (>40) from relatively low amounts of genomic DNA [49] and is gaining popularity due to its simplicity, fast execution, cost efficiency, and robustness.

### 1.5. Implications of CNVs

Significant human inter-population variations in gene copy number, as reported by Redon et al. [6] and Jakobsson et al. [50], suggest that CNVs may be involved in adaptation to various environments, evolution, and susceptibility to common diseases.

In several organisms, a large number of CNVs have been reported in genes with tissue-specific expression rather than in genes that are widely expressed and may have housekeeping activities. The evolution of myoglobin, hemoglobin, trichromatic vision, and olfactory genes are a few of the most often mentioned instances of evolutionarily significant CNVs in humans that conform to this concept. The amylase (AMY) gene family, an enzyme that digests starch, can be used as a multifaceted example in understanding evolutionary processes mediated by CNVs. The number of copies of the AMY genes in modern humans differs from those in other primates and even other species of early humans. The current human population has up to 20 copies of the alpha-amylase 1 gene (AMY1) [51], unlike Neanderthals, who had only two copies. Given that gene expression is affected by the number of its copies and that the copy number variation of the AMY1 gene has been linked to diet, it is an example of recent human evolutionary adaptation. This suggests that our lineage evolved specific adaptations to digest foods rich in starch, foods of increasing importance in our diet [52].

CNVs and other variations of the human genome play an important role in human health and disease. Considering that CNVs occur throughout the genome and can cover a large number of genes and regulatory regions, pathogenic CNVs have been associated with genomic disorders and syndromes as well as complex multifactorial diseases including neurodevelopmental, neurodegenerative, autoimmune, and cardiovascular diseases [16].

There is a common basis and high similarity in the mechanisms through which CNVs can cause disease and yet contribute to evolution. Given that copies of redundant genes can acquire new roles, duplications (or multiplications in any number) are the most commonly mentioned mechanisms considered as key sources of evolutionary variation. If fitness is not compromised because the duplicated gene is not dosage-sensitive, one copy of a gene may retain its original function while the other copy escapes selective pressure, continuously undergoes mutation, and can even develop a new and different function [16].

CNVs also act on evolution and disease through other processes [35], including:

(i) direct influence on the expression of a gene product, giving rise to changing levels of a protein. For example, Miller et al. [53] demonstrated an almost perfect correlation between the α-synuclein (SNCA) gene dosage and its mRNA and protein levels in Parkinson disease. SNCA triplication resulted in a doubling in the effective load of the normal gene and increased deposition of aggregated forms of the protein level in the brain into insoluble fractions.
(ii) alteration of regulatory regions due to CNVs on non-coding sequences. This directly influences the levels and timing of expression and the cellular localization of the related protein. For example, the regulation of SOX9 gene expression in the testis is governed by a set of regulatory elements (RevSex and XYSR) located upstream of its promoter [54,55]. Loss of one or both of these regions in an XY individual results in a loss of SOX9 expression and male-to-female sex reversal [55], while duplication of the RevSex region in an XX individual could increase SOX9 expression and lead to female-to-male sex reversal [56,57,58,59].
(iii) recombination of functional domains of different genes, leading to the formation of modified or new products with newly acquired functions, as seen in the example of glucocorticoid-remediable aldosteronism (GRA). Some researchers have shown that it is caused by a chimeric 11 β-hydroxylase (CYP11B1)/aldosterone synthase (CYP11B2) gene formed when a gene duplication resulting from unequal crossing over fuses the 5′ regulatory region of 11/β-hydroxylase to the coding sequences of aldosterone synthase [60]. The ectopic expression of CYP11B2 in the adrenal zona fasciculata may be responsible for these abnormalities because the gene is normally only expressed in the adrenal zona glomerulosa [35,60].

### 1.6. CNVs and Cancer

Cancer refers to a group of diseases characterized by the uncontrolled proliferation of certain cells in the body with the possibility of invasion or spreading to other parts of the body. The uncontrolled proliferation is due to dysregulation in the activity and expression of genes that control this function [61]. Somatic or germline mutations in tumor suppressor genes and oncogenes are the most well-known causes of cancer. With the increasing use of whole-genome techniques, somatic and germline CNVs have also been recognized as genomic alterations that lead to cancer development [5].

Germline CNVs are present in egg or sperm cells and can be passed down from parent to offspring. If they involve particular genes, an individual can be significantly predisposed to inherited cancers [35] as a result of alterations in DNA repair processes [20,62] or variations in the gene dosage of oncogenes and tumor suppressor genes [63]. Using a hereditary cancer panel to detect cancer susceptibility, Genekor’s Medical S.A. laboratory evaluated a total of 2163 patients [62]. Of these, 1785 had breast cancer, 267 had ovarian cancer, and 111 had colon cancer. NGS and MLPA techniques revealed 464 samples (21.5%) to have pathogenic/likely pathogenic variants (P/LP), referring to alterations in DNA that are predicted to result in a known genetic condition, of which 10.8% (50/464) were attributed to CNVs. Notably, CNVs accounted for 10.2% (37/362) and 6.8% (5/74) of pathogenic variants in breast and ovarian cancer patients, respectively. Meanwhile, in colorectal cancer patients, CNVs were responsible for 28.6% (8/28) of P/LP variants. Out of the 50 CNVs found, 8% were in a low-risk cancer gene (8% FANCA), 20% in moderate-risk genes (4% ATM, 16% CHEK2), and 72% in high-risk genes (2% BRCA2, 8% MSH2, 8% PMS2, and 54% BRCA1) [62].

Somatic CNVs are those present only in particular cells and are primarily non-hereditary. They are acquired during an individual’s lifespan, mostly as a result of environmental factors or errors in cell division. Somatic CNVs are classified as either large-scale variants or focal variants based on their size. Both types are important in the context of disease, but focal variants are considered more suitable for identifying candidate driver genes due to their relatively small size and low gene content [16]. Genome-wide analysis using high-resolution SNP arrays is currently being used to define the extent of somatic CNVs in cancer genomes. This has enabled the observation of a more immediate and direct role of these CNVs in the cancer cells themselves, whereby the cancer cells often display differential gene expression, especially of oncogenes and tumor suppressor genes [64].

Common cancer CNVs. In addition to phenotypic influence, CNVs that are common in the healthy population are also likely to play a role in carcinogenesis. In one correlation study between common CNVs and malignancy [5], all known CNVs in the normal human genome whose loci coincide with those of cancer-related genes such as ERBB2 and TP53 (as cataloged by [65]) were mapped and named common cancer CNVs. Although all gene regions are usually thought to be little affected by CNVs [6], it was surprising that 49 cancer-related genes were found to be directly overlapped or encompassed by a CNV in many individuals from the large reference population of 770 healthy genomes [5]. Each of the common cancer CNVs only slightly increases the risk of disease, but collectively, they can induce a significantly elevated risk [5].

Rare cancers are CNVs. These are the rare CNVs (with a population frequency of <1%) observed in cancer-related genes. Most are associated with hereditary cancer syndromes and involve genes such as FANCA in Fanconi anemia A, CHEK2 in familial breast cancer, RB1 in familial retinoblastoma, and MSH6 in hereditary non-polyposis colorectal cancer [5,66]. There are more than 200 cancer syndromes, most of which arise infrequently, and they account for approximately 5–10% of all cancers [67]. Rare cancer CNVs are often highly penetrant on their own, exhibit autosomal dominant inheritance, and will most often show co-segregation with the disease in families in contrast to low-penetrance alleles [67].

## 2. CNVs and Pancreatic Ductal Adenocarcinoma

Although mutations are recognized as the most commonly known genetic altera-tions able to cause cancer, genomic alterations such as CNVs are also playing an emergent role. Here we focus on the implications of CNVs in PDAC.

### 2.1. Mechanisms of Pancreatic Cancer Pathogenesis

Whole-genome sequencing has revealed that somatic mutations in oncogenic genes such as KRAS and loss-of-function mutations in tumor suppressor genes such as TP53, CDNK2A, and SMAD4 are the main drivers of PDAC [68]. Other causes of PDAC include (i) epigenetic modifications, which in turn lead to altered transcriptional reprogramming; and (ii) chromosomal alterations [69].

**Figure 2:**
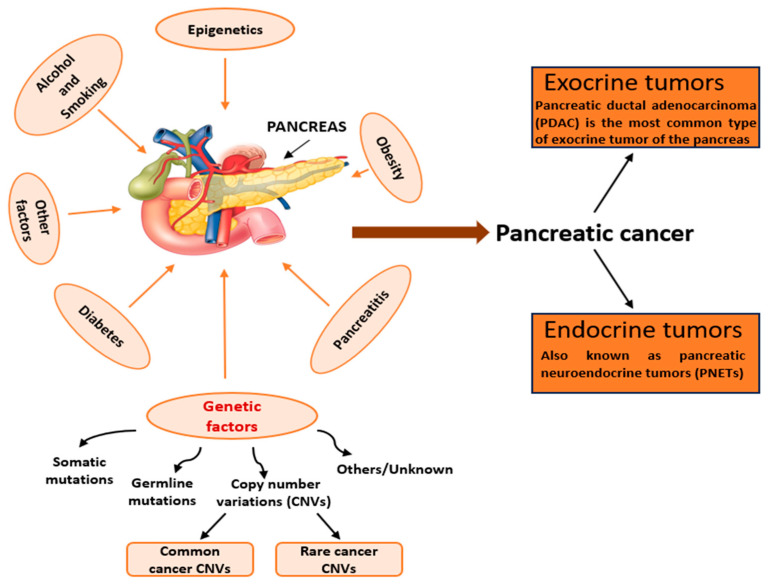
Associations in the development of pancreatic cancer, including the relationship between PDAC and copy number variations (CNVs).

While most PDACs arise sporadically, up to 10% occur in patients with familial and hereditary predispositions. For instance, patients are more likely to develop PDAC if they have germline mutations in the BRCA1, BRCA2, PRSS1, or mismatch repair genes [70]. Even though initial correlation studies showed no significant association between CNVs and PDAC tumorigenesis and progression [71], current research has revealed associations between sporadic and familial pancreatic cancer (FPC) with CNVs [72,73] (Figure 2).

*CNVs in sporadic pancreatic cancer.* CNV analysis in PDAC has revealed common cancer CNVs, including amplifications of KRAS (12p12.1), GATA6 (18q11.2), MYC (8q24.2), ERBB2 (17q12), PAK4 (19q13), NCOA3/AIB1 (20q13.12), SKAP2/SCAP2 (7p15.2), and AKT2 (19q13), as well as deletions of SMAD4 (18q21.2), CDKN2A (9p21.3), CDKN2B (9p21.3), PTEN (10q23.31), MAP2K4 (17p12), RUNX3 (1p36.11), TP53 (17p13.1), DCC (18q21.1), and ARID1A (1p36.11) [74,75,76]. Other CNVs are reported in Supplementary Table S1. However, it has not been established whether these CNVs are the cause or effect of cancer.

*CNVs in familial pancreatic cancer.* Hereditary CNVs in the genome may also contribute to a genetic susceptibility to PDAC. One study used representational oligonucleotide microarray analysis (ROMA) to characterize germline CNVs in 60 cancer patients from 57 FPC families, i.e., those in which at least two first-degree relatives have been diagnosed with pancreatic cancer. A total of 56 distinct genomic areas, including 25 deletions and 31 amplifications, were found to have CNVs that were not present in the healthy controls [77]. Among these CNVs, functionally interesting candidate genes were selected whose germline amplification (e.g., JunD, MAFK, RND1, WNT10B, WNT1, MAP2K2, and BIRC6) or deletion (e.g., ANKRD3, PDZRN3, and FHIT) may contribute to tumor development. These CNVs may define potential candidate loci for familial PDAC.

### 2.2. Identification and Analysis of CNVs in PDAC

Overexpressed proteins in PDAC due to CNVs could be therapeutic targets as well as diagnostic and prognostic markers. For example, in a genome-wide analysis of 27 microdissected PDAC samples using high-density microarrays representing ∼116,000 single nucleotide polymorphism (SNP) loci, frequent gains of 1q, 2, 3, 5, 7p, 8q, 11, 14q, and 17q (≥78% of cases) and losses of 1p, 3p, 6, 9p, 13q, 14q, 17p, and 18q (≥44%) were detected [76]. Quantitative real-time PCR revealed that the SKAP2 gene (7p15.2), a member of the src family kinases, was the most frequently amplified (≥3 copies found in 59–63% of cases), and reverse transcription PCR was used to confirm its recurrent overexpression in eight out of 12 PDAC cases (67%). Moreover, in situ RNA hybridization (ISH) and FISH analyses revealed a significant correlation between SKAP2 DNA copy number and its mRNA expression level, suggesting that SKAP2 upregulation is due to CNVs [76,78]. The overexpression of SKAP2 was observed consistently from early-stage (I–II) to late-stage (III–IVb) tumors, suggesting a potential involvement of this gene in the development of PDAC, including control of the growth and differentiation of PDAC cells via α-Synuclein [79], as well as modulation of their motility and spread by interacting with the focal adhesion kinase RAFTK [80]. Based on these findings, scientists proposed that the SKAP2 gene could be used as a potential target for therapeutic intervention as well as a potential marker gene for early diagnosis in PDAC [76].

GATA binding protein 6 (GATA6), a zinc-finger transcription factor that plays an important role in the normal development of endodermal and mesodermal tissues, including the pancreas, is amplified in PDAC due to CNVs [81]. Since the progression of normal pancreatic ductal epithelium to infiltrating cancer is believed to occur through a series of morphologically defined precursors known as pancreatic intraepithelial neoplasia (PanIN-1, 2, and 3) [82], the GATA6 copy number was assessed in microdissected samples of normal duct epithelium, PanIN, and human PDAC to investigate its role in PDAC. Quantitative PCR revealed no gain of GATA6 in normal duct epithelium (0 of 4), PanIN-1 (0 of 13), or PanIN-2 (0 of 10) lesions when compared to the haploid genome [83]. However, an increased GATA6 copy number (≥2.3 copies) was identified in 6/17 samples (35%) of PanIN-3 and in 18/55 samples (33%) of PDAC, and confirmed through FISH in paraffin-embedded sections of 10 PDAC samples and one PanIN-3. This GATA6 amplification and consequent transcriptional upregulation observed late in PDAC carcinogenesis suggest that detectable GATA6 copy number gain may have value as a diagnostic marker [83]. Early findings from Comprehensive Molecular Characterization of Advanced Pancreatic Ductal Adenocarcinoma for Better Treatment Selection (COMPASS; a prospective study: NCT02750657) further demonstrated that molecular profiling can predict how different patients with locally advanced or metastatic PDAC and with different genomic and transcriptome subtypes will respond to chemotherapy. Patients with the transcriptomic “basal-like subtype”, a highly chemoresistant phenotype, have a shorter median overall survival than those with the “classical” subtype. The latter are easily identified by positive GATA6 staining by an RNAscope in situ hybridization (ISH) assay and high GATA6 expression. GATA6 could therefore be a useful marker for the classical subtype [84,85].

Another target of gene amplification in PDAC is MYC, a member of a family of transcription factors that work together to control cell proliferation, metabolism, and the expression of genes necessary for these processes [86]. Pre-clinical experimental evidence has shown that MYC is an essential and non-redundant node of oncogenic signaling and therefore should be a therapeutic target [87,88,89,90]. It is usually upregulated by gene amplifications, and consequently, it can enhance the progression of cancer by promoting cell competition, survival signals in hypoxic settings, and altered metabolic pathways. This amplification is inversely correlated to that of GATA6, and a high MYC expression level is typical in the basal-like PDAC subtype [91]. MYC also has an emerging role in remodeling the tumor microenvironment (TME). TME is a distinctive feature of PDAC that makes up about 90% of the tumor mass and is characterized by a prominent desmoplastic reaction [92]. MYC amplification in PDAC induces the depletion of CD3 T cells while increasing the recruitment of immune cells such as neutrophils, macrophages, B cells, and granulocytic myeloid suppressor cells [93], collectively enhancing an immunosuppressive phenotype [93,94]. Genomic and transcriptomic analyses have further linked MYC to a high number of metastases in patients (>10 metastases in a patient) in PDAC [95]. In terms of drug resistance, MYC overexpression has been linked to the resistance to inhibitors of the serine/threonine protein kinase mammalian target of rapamycin (mTOR) [96,97,98,99,100,101].

Alteration of regulatory regions due to CNVs on non-coding sequences can also influence the level and timing of expression of the related protein [102]. In a case–control cohort consisting of 1031 controls and 1027 pancreatic cancer cases, researchers demonstrated that CNVR2966.1, a CNV located in a gene desert region on 6q13, is significantly associated with the risk of developing disease and functions as a potential trans-acting regulator of the CDKN2B (p15 or INK4B) gene located on 9p21.3. CNVR2966.1 is an insertion/deletion and chromosome conformation capture-on-chip (4C), and other functional experiments have shown that it may contain a transcriptional activation element and regulate CDKN2B transcription through interchromosomal long-range interaction. CDKN2B is a tumor suppressor that encodes a cyclin-dependent kinase inhibitor that regulates cell growth and the cell cycle G1 progression by preventing the activation of cyclin-D-dependent kinases [103]. It has been found to be frequently co-deleted with the neighboring tumor suppressor gene CDKN2A (which codes p16-INK4a and p14ARF) in various tumors, and its deletion has been reported in a significantly high proportion in pancreatic cancer [76,104]. Therefore, CNVR2966.1 may be important for risk assessment, early detection, and a better understanding of PDAC [72].

In another study aimed at exploring potential biomarkers of PDAC, analysis of transcriptomic and clinical data from The Cancer Genome Atlas Program (TCGA) revealed high expressions of the COL17A1 and ECT2 genes and associated this expression with CNVs [105]. The highly expressed genes of these patients were also related to the cell cycle and proteasome pathways. COL17A1 is a transmembrane protein that can affect the proliferation and differentiation of epithelial cells and therefore acts as an important factor in the formation and maintenance of multilayered epithelial structures in PDAC [106], while ECT2 is an oncogene that plays an important role in cell proliferation and metastasis. Clinical correlations further showed that the expression of these two genes was significantly associated with tumor grade and that the overall survival (OS) rate decreased with an increase in their expressions. Since several research studies have demonstrated the success of combining anti-PD-1 antibody immunotherapy with chemotherapy in treating PDAC [107,108], this study further demonstrated that the high-ECT2 group exhibited greater sensitivity towards anti-PD-1 therapy and 20 chemotherapeutic agents (e.g., bortezomib and rapamycin). These discoveries suggest that ECT2 and COL17A1 are potential diagnostic and prognostic markers for PDAC that can also facilitate innovative approaches for personalized treatment [105].

### 2.3. CNV-Based Classifications of PDAC

Researchers proposed that reclassifying PDAC into subtypes based on genetic and molecular characteristics may guide novel treatment choices with prognostic and biological significance [109]. According to this hypothesis, PDAC has further been classified into structural [75] and molecular [110] subtypes, for example, based on CNVs as highlighted below.

#### 2.3.1. Structural Variation Profiles

Some researchers performed a deep WGS and CNV analysis using SNP arrays in 100 normal and tumor-derived samples obtained from patients with PDAC. After retrieval, validation of the presence of carcinoma in the samples to be sequenced, and estimation of the ratio of malignant epithelial nuclei to stromal nuclei, the samples were removed, followed by processing in formalin or full-face sectioning using optical coherence tomography (OCT). Macrodissection was carried out, when necessary, to excise non-malignant tissue areas, followed by the extraction of nucleic acids. CNVs analyses led to a classification of the disease into four subtypes based on the number, frequency, and distribution of structural rearrangement events across the genome in each patient [75]. The majority of these structural rearrangements were due to a copy number change (events classified as deletion, duplication, tandem duplication, amplified inversion, and foldback inversion).

*Stable (subtype 1).* Tumors contain a few structural rearrangements (<50) located randomly throughout the genome. They exhibit aneuploidy, suggesting cell cycle/mitosis defects, given that although aneuploidy was classically defined as whole chromosome numerical aberrations, this definition has recently been expanded in the cancer genome literature to include losses or gains of chromosome arms [111,112,113].

*Locally rearranged (Subtype 2).* Tumors exhibit non-random intra-chromosomal rearrangements on one or a few chromosomes. These are further classified as either: (i) focal amplifications where most of the events are gains in known oncogenes including GATA6, SOX9, and KRAS, as well as therapeutic targets like CDK6, MET, ERBB2, PIK3R3, and PIK3CA; or (ii) complex rearrangements involving complex genomic events like breakage–fusion–bridge (BFB) or chromothripsis (i.e., the simultaneous occurrence of multiple structural alterations in a single mitotic event) [114].

Although in this subtype the most known oncogene copy-number increases in tumors were observed in a few patients, most of these oncogenes are well-recognized therapeutic targets (MET, FGFR1, ERBB2) with readily available inhibitors. The other oncogene amplifications identified include GATA6, which is known to be amplified in PDAC and correlates with a poor prognosis [75].

*Scattered (Subtype 3).* Tumors contain 50–200 structural rearrangements scattered throughout the genome.

*Unstable (Subtype 4).* Tumors contain many structural rearrangements (>200) scattered throughout the genome. Such a large scale of genomic instability suggests defects in DNA maintenance, in addition to potentially highlighting sensitivity to DNA-damaging agents.

Notably, these authors did not perform clinical correlation analyses.

#### 2.3.2. Molecular Subtypes

Some researchers profiled genomic alterations in a Chinese cohort of 608 PDAC patients from a database containing somatic mutations, CNVs, and pathogenic germline variants [110]. Targeted-region capture and sequencing were performed using two gene panels specifically designed for cancer gene detection, comprising 566 and 764 genes, respectively. Germline and somatic CNVs were identified, and this information was used to perform unsupervised consensus clustering of the patients as well as differential CNV analysis. Functional/pathway enrichment analysis was then conducted for genes with significantly higher CNV values in each cluster or group. More specifically, consensus clustering revealed two groups, namely CNV-G1 and CNV-G2. Based on the CNV of genes involved in DNA repair and receptor tyrosine kinase (RTK)-related signaling, patients from CNV-G1 were further subdivided into two subtypes: the proliferation-active subtype and the repair-deficient subtype. Patients from CNV-G2 were also subdivided into two subtypes: the repair-enhanced and the repair-proficient subtypes [110].

CNV-G1 is characterized by deletions predominantly in DNA repair genes, higher copy number instability (CNI), and defects in DNA-DSB (double-strand break) repair by homologous recombination (HR). It consists of the (i) proliferation-active group with a high CNV score and amplification of genes in the RTK-related signaling pathway, and the (ii) repair-deficiency group with a low CNV score.

CNV-G2 is characterized by amplifications predominantly in DNA repair genes, a higher tumor mutational burden (TMB), and defects in polymerase POLE. It consists of the (i) repair-enhanced group with a low CNV score and amplification of genes in the HRR pathway, and the (ii) repair-proficient group with a high CNV score.

The prognosis of the repair-deficient subtype was better (median survival time of 410 days) than that of the other three subtypes, suggesting that deletion of genes in the DNA repair pathway (specifically the HRR pathway) causes greater genomic instability and is detrimental to the survival of cancer cells. On the contrary, patients in the proliferation-active and repair-enhanced subtypes showed worse prognoses, with median survival times of 197 and 239 days, respectively. Furthermore, the prognosis of the proliferation-active subgroup was worse than that of the repair-deficient subgroup, suggesting that genetic amplification in RTK-related signaling would promote cancer cell proliferation and thereby confer a worse prognosis [110].

Together with the evidence from genomic footprint analysis, the study proposes that repair-proficient and repair-enhanced subtypes are better suited for immunotherapy, while DNA-damage therapies (such as platinum-based chemotherapy and PARPi) are highly recommended for repair-deficient and proliferation-active subtypes [110].

## 3. CNV Studies in PDAC

### 3.1. Literature Review

**Figure 3:**
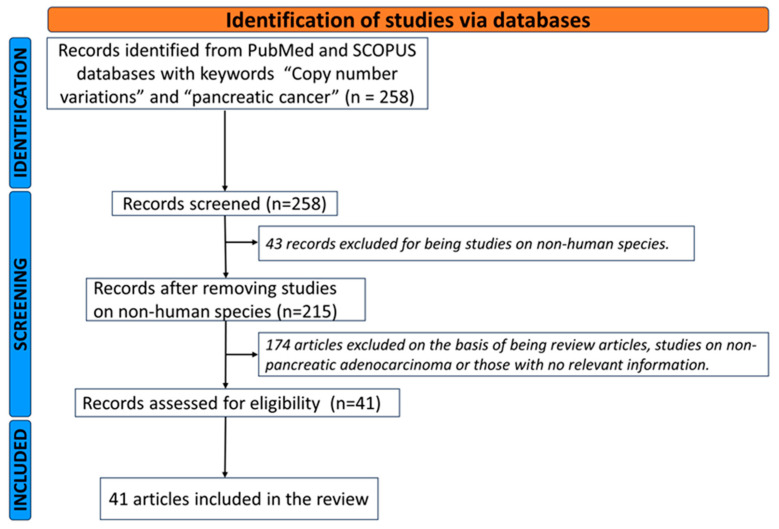
A flow chart of the identification process of the included articles: 258 papers were initially identified by title, of which 41 fulfilled the inclusion criteria after full-text evaluation. A total of 41 papers were included.

We carried out a literature search for several published papers on copy number variations and pancreatic cancer and highlighted the CNV landscape in the disease (Supplementary Table S1). We analyzed a total of 41 published articles from PubMed and SCOPUS in which researchers examined the expression levels of the genes that were discovered to have amplifications or deletions (in pooled public datasets or samples) in normal pancreatic tissues in comparison to malignant tissues (Figure 3). We further analyzed the biological and clinical importance of these studies, particularly whether these genes displayed dysregulated expression linked to survival outcomes.

Samples were obtained from diverse sources, with most studies being carried out on samples from primary tumors only (21/41). Other studies, however, included both primary and metastatic samples (5/41). Other sample sources included were from public databases such as TCGA and NCBI GEO (13/41), tissue microarrays (TMAs) samples (1/41) and peripheral leukocytes from patients and controls (1/41). Some studies included cell lines (8/41) in the verification of identified CNVs, while others (33/41) did not.

Most of the studies were performed using “whole” (whole genome or whole exome) approaches (63.5%), including SNP-arrays (10/41), aCGH (8/41), tissue microarrays (TMA) (2/41), NGS (4/41) and both SNP/aCGH (2/41) techniques. Targeted approaches were used in the rest of the studies we considered (36.5%). Use of the “whole” approach resulted in the identification of numerous CNVs in the whole PDAC genome in comparison to normal controls; however, subsequent studies such as the roles of the identified CNVs in the development and progression of disease as well as their effects on currently available therapy were focused only on a few selected genes.

We noted that the use of diverse techniques for analyzing CNVs in different PDAC samples, as well as confirming their effect on levels of mRNA expression, did not significantly affect the consistency of the results. Moreover, most of the CNVs detected could be verified in various publicly available datasets, such as NCBI GEO and The Cancer Genome Atlas Program (TCGA). Recurrent gains on chromosomes 1q, 2p, 3q, 5p, 6p, 7q, 8q, 11q, 12p, 15q, 17q, 18q, 19q, and 20q included several known or suspected oncogenes, and recurrent losses on chromosomes 1p, 3p, 6, 8p, 9p, 10q, 12q, 13q, 15q, 17, 18, 19p, 20p, 21 and 22, which included several known or suspected tumor suppressor genes. In general, tumors with more copy number alterations (an indicator of chromosomal instability) trended toward a poor prognosis.

As expected, CNVs were almost always observed in the classical mutation genes in PDAC, including amplification of the oncogene KRAS (12p12.1) and deletions of the tumor suppressor genes TP53 (17p13.1), CDNK2A (9p21.3), and SMAD4 (18q21.2) to further confirm their role in the disease [75,76,115,116,117,118,119,120,121,122,123,124,125,126,127,128,129]. Interestingly, the frequencies of CNVs are consistent throughout various ethnicities, even though disparities have been observed in the frequency of driver mutations in PDAC, such as a lower frequency of KRAS mutations in Korea [130,131] and Japan [125]. For instance, one study performed microarray and CNV analyses of 93 pancreatic cancer data derived from the Japanese version of the Cancer Genome Atlas (JCGA) and revealed frequent CNVs as gains in 3q, 7q, and 2q and losses in 7q, 12q, 19q, and 19p [125], which are consistent with CNVs in other ethnicities [75,76,115,116,117,118,119,120,121,122,123,124,125,126,127,128,129].

The most frequent CNVs reported were amplifications of MYC (8q24) (15/41) and GATA6 (18q11.2) (7/41), and deletions of CDKN2A (9p21.3) (16/41), CDKN2B (9p21.3) (7/41), and SMAD4 (18q21.2) (14/41). MYC overexpression is typical in the basal-like PDAC subtype, which exhibits poor prognosis and chemoresistance. GATA6 overexpression is typical of the classical PDAC subtype, and its expression is observed late in PDAC carcinogenesis, suggesting that detectable GATA6 copy number gain may have value as a diagnostic marker. GATA6 overexpression has been associated with poor prognosis, but interestingly, it has been shown to correlate to a better prognosis after resection and adjuvant therapy, where it was believed to act as a suppressor of mutant KRAS^G12V^-driven PDAC [75,117,121,123,126,132,133].

In chromosome 1, the amplifications of 1p12 (NOTCH2) [115,118,123] and 1p13.1-p12 (REG4) [75,116,117] and the deletion of 1p36.11 (ARID1A) [75,117,126] were the more recurrent CNVs. REG4 overexpression was associated with poor prognosis and resistance to gemcitabine treatment in one study, suggesting that adjuvant therapies that target reg4 could enhance the usual gemcitabine-based treatment of pancreatic cancer [75,116,117].

ASAP2 (2p25.1) amplification has been associated with lower overall survival (OS) as well as lower relapse-free survival (RFS) [134,135]. FHIT (3p14.2) and ATR (3q23) deletions were mostly reported in both sporadic familial cases, indicating their possible role in PDAC susceptibility as well as progression [77,110,118,133,136]. FHIT (3p14.2) deletions and ECT (3q26.31) amplifications have also been correlated with poor prognosis in PDAC [73,77,105,118,133,136].

The most recurrent amplification on chromosome 8 was 8p11.21 (FGFR1, IDO1, ZNF703) [110,123,126,129], observed in both sporadic and familial PDAC. One study demonstrated that the loss of 8p was exclusively observed in patients with shorter survival and associated this with specific CNV acquisitions due to potential positive selection and genetic drift. The genes that have been associated with this location are 8p, 8p23.2 (CSMD1) in sporadic PDAC, 8p23.1 (MCPH1 and ANGPT2), and 8p22 (NAT1) in FPC [116,117,136].

Some researchers examined a patient’s complicated evolutionary history and very long postsurgical survival period (43 months) and proposed that this could be due to the amplification of a segment 9p.22 covering the FREM1 gene, which has recently been linked to increased immune cell (IC) infiltration. They suggested that an active immune response could improve the outcome. FREM1 in this particular context could be further explored both as a molecular target and/or immune checkpoint-blocking therapeutic strategy and as a biomarker of an active local immunological response [116].

No deletions were reported on chromosome 11, but there were amplifications on 11q13.3 (CCND1, TMEM16H), 11q13.5 (EMSY), and 11p14.1 (LGR4), of which the CCND1 gene was the most recurrent amplification [118,120,132,134,137]. One study showed that the presence of elevated EMSY copy numbers in relatively large, clustered cells surrounded by tumor cells expressing normal copy numbers suggests that the mutation occurred later in the carcinogenesis process rather than at an early stage. This could clarify a previous investigation that discovered a negative correlation between this mutation and the course of the disease [137].

In one study, TMEM132E (17q12) amplification was prevalent in a relapse (within 1 year after resection) subgroup (*n* = 15) compared with a non-relapse subgroup (*n* = 15) of 47% vs. 7% [138].

Some researchers demonstrated that the loss of a specific cytoband, 18q22.3, which encompasses only five genes, including the carboxypeptidase of glutamate-like (CPGL) gene, is linked to a poorer prognosis in both a testing cohort and an independent validation cohort of surgically resected pancreatic cancers. Further experiments involving reintroducing the CPGL gene, or its splicing variant CPGL-B, into CPGL-deficient pancreatic cancer cells showed a reduction in anchorage-independent cell growth and migration while promoting G1 accumulation. These findings imply that CPGL is a novel growth suppressor for pancreatic cancer cells and that risk classification in pancreatic cancer patients who have had their tumors removed could be based on the CPGL gene [132].

In one study, the perineural invasion in PDAC was linked to gains of 4q13.3, 4q35.2, 7p12.2, 10q26.3, 11q13.3, 17q23.1, 22q13.32, and loss of 6p21.32, whereas the amplification of 8q24.13 was strongly correlated with the T, N, and M stages simultaneously [134].

Some CNVs have also been associated with the increased glycolysis observed in PDAC. One study compared the SNP microarray data of glycolysis-high samples to glycolysis-low samples and found substantial amplifications of MYC (8q24.2), GATA6 (18q11.2), FGFR1 (8p11.21), and IDO1 (8p11.21), as well as deletions of SMAD4 (18q21.2) that were associated with the aerobic glycolysis phenotype characteristic of PDAC [123].

Another study demonstrated that the assessment of overall CNV burden through genome-wide methylation profiling could be a valuable prognostic tool in patients with surgically treated PDAC [129]. By analyzing DNA extracted from 108 chemotherapy-naïve, surgical PDAC specimens, the researchers were able to gather data on the DNA methylation status of more than 850,000 CpG sites located in various regions such as the promoter, enhancer, and gene body. Morphological subtyping, as per Kalimuthu et al. [139], classified PDAC into Group A tumors, which showed a dominant conventional and/or tubulopapillary growth pattern, and Group B tumors, which showed a dominant composite and/or squamous growth pattern. CNV profiles were then generated from the accumulated CpG methylation signal distributed throughout the genome (except for 13p, 14p, 15p, 21p, and 22p, X, and Y), and all the PDACs were classified into three distinct groups based on the number of chromosomal arm-level alterations: high (≥17), moderate (5–16), or low (0–4). The most prevalent chromosomal arm-level aberrations included gains of 1q (19%) and 8q (29%), as well as losses of 8p (25%), 19p (26%), 6p (26%), 9p (36%), 6q (37%), 18q (43%), and 17p (55%). In particular, the CNVs involved deletions of CDKN2A/B, KDM6A, and SMAD4 and focal amplifications of MYC, FGFR1, or CDK6. Overall, low CNV burden was observed in Group A tumors, while high CNV burden was observed in Group B tumors, and this higher CNV burden in Group B was further associated with a poor prognosis and shorter overall survival [129]. Notably, this study was performed on PDAC-enriched FFPE tissues, and further studies are necessary to establish the possibility of performing CNV burden analysis on endoscopic ultrasound-guided fine-needle biopsies from non-resectable PDAC patients, as well as whether this has any prognostic value [140,141].

In another study, by analyzing 21 FFPE tumor tissues of PDAC patients, the authors analyzed the mutational spectrum of the disease and assessed the therapeutic relevance of OncoPan, a previously developed and validated NGS panel of 37 genes [142]. This panel includes the evaluation of indels, SNVs, and CNVs of various actionable genes for the identification of therapeutic targets as well as inherited cancer syndromes. Oncopan led to the discovery of biomarkers for personalized therapy in five PDAC patients. Among these patients, two exhibited HER2 amplification, making them potentially eligible for immunotherapy [142]. Numerous ongoing clinical studies are utilizing trastuzumab for pancreatic cancer treatment, and in a recent study, Hirokawa et al. reported that patients with HER2-positive heterotopic pancreatic cancer responded well to trastuzumab treatment [143]. These kinds of studies are a practical example of the clinical relevance of CNVs, and therefore it is expected that further/new panels will be developed to evaluate CNVs of a greater number of genes.

In the future, these assessments will also be less invasive thanks to the possibility of carrying out liquid biopsies. In fact, the use of circulating tumor DNA (ctDNA) is gaining significant popularity in molecular diagnosis, observation of clonal evolution, evaluation of treatment response, identification of cancer recurrence, and evaluation of drug resistance [144,145,146]. One study in 48 late-stage non-small cell lung cancer (NSCLC) patients analyzed matched tumor tissues and blood samples and determined gene-level CNVs from ctDNA [147]. Although the identification of somatic CNVs from ctDNA samples using targeted sequencing is challenging, amplifications of the EGFR, ERBB2, and MET genes were observed. Further comparison of these amplifications between tissue WES and ctDNA showed significantly high concordance and sensitivity, with 100% specificity observed for all three genes. Although the study was performed on NSCLC, the pipeline can be extended to other cancers, including PDAC [147], where liquid biopsies sequencing provides an alternative to obtaining the patient’s genomic information in cases where tissue biopsies are not available [146].

### 3.2. CNVs in PDAC Stages and Grades

Research is being carried out to identify CNVs that may be useful clinical markers, and a recent study on ovarian cancer has provided results that encourage continued in-vestigation of these relationships. In particular, CNV-profiling analyses have been suc-cessfully used to distinguish between malignant and nonmalignant, as well as early and late stages in ovarian tumors [148]. The possible roles of CNVs in the early or late stages of pancreatic cancer have also been studied to assess their usefulness as potential markers of the various stages as well as the grades of the disease. For example, a gain in copy number at the 7p15.2 locus that causes the overexpression of the SKAP2 gene characterizes both PanIN lesions and early- (I–II) and late-stage (III–IVb) PDAC tumors. Therefore, it could be a potential marker gene for early diagnosis as well as a possible target for therapeutic intervention [76].

Some researchers also studied the relationships between 19q13 amplification and clinicopathological characteristics in PDAC and observed that the frequency of 19q13 gains increased from G1-G2 (low/moderate) to G3 (high grade) tumors and from pT1-pT2 (early) to pT3-pT4 (late) stage tumors. Moreover, none of the G1 tumors exhibited 19q13 copy number changes, while 11% of G2 tumors and 16.8% of G3 tumors displayed an in-crease in 19q13 copy number [149].

Among the CNVs in PDAC, amplification and overexpression of the PSCA and HMGA2 genes have further been associated with lymph node metastasis (N0) and invasive depth of the disease, respectively [124].

In another study, SNP arrays on 20 PDAC tumors identified two different CNV groups with different genetic profiles: group 1 (*n* = 9) showed losses at Xp22.33, 17p13.3, 9p24.3, 9p22.1, 6q25.2, and 1p36.11 chromosomal regions and gains at 1q21.1, while group 2 (*n* = 11) showed gains at 22q13.32, 22q13.31, 22q13.1, 16q24.3, 16q24.1, 11q13.4, 11q13.3, 11q13.1, 10q26.3, 10q26.13, 5q32, 3q22.1, and 2q14.2 chromosomal regions. From a clinical and histological perspective, grade I/II PDAC tumors that were smaller and well- or moderately-differentiated were linked to group 1 cases, while grade III carcinomas that were primarily poorly-differentiated made up group 2 PDAC cases, which were bigger in size [150]. Further analyses of these CNV regions showed that they harbor various cancer-associated genes, including those that have been specifically associated with PDAC, such as the TNFRSF6B gene, whose amplification has been observed in many tumors [151,152,153,154] and whose overexpression is known to block growth inhibition signals in PDAC [155], and the MAPRE2 gene, whose deletion has been observed in leukemic cells [156] as well as pancreatic cancer [157]. In this study, deletions of other genes, such as MYOCD [158] and PTAFR [159], were found to be recurrent in PDAC, although the association of these genes with PDAC pathogenesis should be further investigated [150].

### 3.3. CNVs in PDAC Chemoresistance

Using aCGH and qPCR in 14 PDAC samples, some researchers detected and confirmed gains in the copy number of the REG4 gene (1p13.1-p12) in all the analyzed samples [160]. CNV analysis in six pancreatic precancerous lesions (PanINs) also revealed an increase in REG4 copy number (in 6/7, 1/7, and 0/6 of PanIN3, PanIN2, and PanIN1 lesions, respectively), suggesting that this amplification is an early event in PDAC development [160]. REG4, a member of the multigenic family named reg, plays a role in the resistance of cells to anticancer drugs like 5-fluorouracil and methotrexate [161,162], and it promotes over-expression of the antiapoptotic proteins Bcl-xL, Bcl-2, and survivin, as well as the phosphorylation of AKT [162,163]. Its overexpression is observed in cancerous tissues of the stomach [164], colon [161,165], and pancreas [166]. In this study, PDAC-derived cells with REG4 protein overexpression grew more rapidly and were more resistant to gemcitabine treatment, and this enhanced growth was also confirmed in PDAC cell lines. Circulating REG4 protein is therefore a potential target to make PDAC sensitive to gemcitabine [160].

## 4. Challenges and Limitations in Clinical Application

Interpretation of any detected CNVs is important because they could have clinical implications [167,168], but this is faced with various challenges. Determining the pathogenicity of CNVs is difficult, and accurate interpretation often depends on the amount of information available in databases [7]. However, there are several important considerations when utilizing public databases. Firstly, there may be variations in the reported sizes of identical CNVs due to the usage of various array platforms [169]. For example, a large number of the previously reported benign CNVs may be overestimated in size because they are based on the bacterial artificial chromosome (BAC) microarray technique [170]. Secondly, it is not always possible to obtain sex information about the individuals included in these databases. This is particularly significant when studying X-linked CNVs in males, as many of the reported benign variants found in the databases are observed in females. However, the same alteration may already be pathogenic in males who possess only one X chromosome. Thirdly, the majority of CNVs reported in large population studies have not undergone validation. Lastly, factors such as incomplete penetrance, variable expressivity, age of onset, and parent of origin imprinting effects were not recorded [7,171].

It is also important to note that the interpretation of CNVs is heavily reliant on the specific clinical indications, and therefore clinicians must provide detailed clinical phenotypes to enable accurate interpretation of the results [172]. To facilitate this process, several groups have devised graphical workflows for CNV interpretation, which prove invaluable in routine diagnostic work. However, interlaboratory comparisons and external quality control schemes (such as the European Molecular Genetics Quality Network (EMQN) and the USA quality assessment scheme CAP (College of American Pathologists)) on the use of some technologies, such as arrays, in diagnostic laboratories show that there are differences in the interpretation, quality, and reporting among laboratories [172]. Therefore, the minimum detection resolution, reporting, and interpretation of CNVs should be standardized among laboratories.

The different types of CNV analysis software used are also unique, frequently employing varying default settings and/or statistical methodologies [173,174,175,176,177,178]. Additionally, each laboratory implements its own experimental and analysis protocols. These variations in protocols and software directly affect the sensitivity and resolution of a test, giving results that are very different from each other or only partially in agreement [172].

## 5. Conclusions

Copy Number Variations (CNVs) are the most frequent genetic structural alterations, making up approximately 12% of the human genome [6].

Currently, many lines of evidence have also shown that CNVs play important pathogenic roles in a variety of human disorders, from causative high-penetrance CNVs in rare genomic disorders to intermediate or low-penetrance CNVs in complex multifactorial diseases such as cancer [16,17,18]. The identification of these amplification and deletion events is therefore one of the main goals of medical genetics research.

Indeed, CNVs have been observed in patients with pancreatic ductal adenocarcinoma (PDAC) [74,75,76,77,179,180,181,182,183,184,185,186,187]. However, the detection of CNVs and their subsequent association with functional and clinical phenotypes remains very challenging. With the increasing use of whole-genome technologies to detect CNVs, germline and somatic CNVs are now recognized as frequent contributors to the spectrum of mutations leading to PDAC development, progression, and drug resistance.

Recent advances in technology have provided powerful tools for the detection and analysis of CNVs at the level of the genome as well as for targeted loci. For example, single-cell RNA-seq (scRNA-seq) studies in human tumors have revealed new insights into tumor heterogeneity and distinct subpopulations, which are pivotal for comprehensively dissecting tumor-related mechanisms [188]. In PDAC, scRNA-seq has been used to acquire the transcriptomic atlas of individual pancreatic cells from primary and metastatic tumors, as well as control pancreases and identify diverse stromal and malignant cell types. This has facilitated the comprehensive delineation of PDAC intratumoral heterogeneity and the underlying mechanisms for PDAC progression [188].

The correlation between CNV and gene expression suggests that the analysis of cancer genome CNVs may be useful in informing therapeutic decisions on the management of individual patients with particular patterns of mutations [109].

Although it is evident that CNVs have a significant impact on inter-individual variation in gene expression, the full extent to which they contribute to the molecular basis of PDAC remains to be established. This is due to persistent technical challenges in the accurate measurement of CNVs [189]. Further studies, using accurate genotyping assays in large population cohorts, will help to define the overall role of CNVs in PDAC pathogenesis more precisely [63].

## Data Availability

Data is contained within the article or Supplementary Material.

## Supplementary Materials

The following supporting information can be downloaded at: https://www.mdpi.com/article/10.3390/ijms25010391/s1.
